# Texture and Color Enhancement Imaging Increases Color Changes and Improves Visibility for Squamous Cell Carcinoma Suspicious Lesions in the Pharynx and Esophagus

**DOI:** 10.3390/diagnostics11111971

**Published:** 2021-10-23

**Authors:** Akira Dobashi, Shingo Ono, Hiroto Furuhashi, Toshiki Futakuchi, Naoto Tamai, Takashi Yamauchi, Machi Suka, Kazuki Sumiyama

**Affiliations:** 1 Department of Endoscopy, The Jikei University School of Medicine, Tokyo 105-8461, Japan; onoshingo@mbe.nifty.com (S.O.); ms04furuhashi@gmail.com (H.F.); futakuchi.t@gmail.com (T.F.); tamai-naoto@jikei.ac.jp (N.T.); kaz_sum@jikei.ac.jp (K.S.); 2 Department of Public Health and Environmental Medicine, The Jikei University School of Medicine, Tokyo 105-8461, Japan; yamauchi-t@jikei.ac.jp (T.Y.); suka@jikei.ac.jp (M.S.)

**Keywords:** superficial esophageal cancer, pharyngeal cancer, squamous cell carcinoma, texture and color enhancement imaging, image enhanced endoscopy, color difference

## Abstract

Texture and color enhancement imaging (TXI) has been developed as an image-enhanced endoscopy technology. TXI mode2 enhances texture and brightness, and TXI mode1 also enhances color. This study aims to assess the color differences in squamous cell carcinoma (SCC) suspicious lesions in the pharynx and esophagus using white light imaging (WLI), TXI mode1, TXI mode2, and narrow-band imaging (NBI). A total of 59 SCC suspicious lesions from 30 patients were analyzed. The color differences (ΔE) between the lesion and the surrounding mucosa were calculated for each modality. The color value was assessed using the Commission Internationale d’Eclairage L*a*b* color space. The visibility of the lesion in each modality was evaluated and compared to that in the WLI by six endoscopists. The mean ΔE values in the WLI, TXI mode1, TXI mode2, and NBI were 11.6; 18.6; 14.3; and 17.2, respectively, and the ΔE values of TXI mode1, TXI mode2, and NBI were significantly higher than those of the WLI (*p* < 0.001). No lesions had worse visibility, and 62.5% (37/59) had improved visibility, as assessed by more than half of the endoscopists in TXI mode1. TXI mode1 can enhance color changes and improve the visibility of SCC suspicious lesions in the pharynx and esophagus, compared to WLI.

## 1. Introduction

The risk factors for SCC in the pharynx and esophagus, which include smoking and alcohol consumption, are common. According to the field cancerization phenomenon, squamous cell carcinoma (SCC) in the pharynx and esophagus occasionally develops synchronously or metachronously [1]. The prognosis of pharyngeal and esophageal SCC is poor when detection occurs at an advanced stage. However, if SCC is detected early, the disease can be cured entirely by local resection. Patients can receive less invasive forms of treatment, such as endoscopic submucosal dissection, and obtain a preferable prognosis with a high quality of life [2,3]. Thus, the early detection of pharyngeal and esophageal cancers is essential.

Most early-stage pharyngeal and esophageal cancers do not have accompanying symptoms, such as choking on food, and are usually found by chance during screening endoscopy. Patients expect endoscopists to detect pharyngeal or esophageal cancer at an early stage. However, it was reported that white light imaging (WLI) sensitivities for superficial esophageal SCC (SESCC) and superficial pharyngeal SCC (SPSCC) were only 55% and 8%, respectively, even with expert endoscopists using high-definition endoscopy [4]. The warning signal for SESCCs or SPSCCs is a subtle color or morphological change in the WLI [5], and it has been challenging to detect malignancies at an early stage, before the development of image-enhanced endoscopy (IEE) [4,5,6,7,8,9,10]. Narrow-band imaging (NBI) systems can enhance microvessels well into the mucosal surface, and both SPSCCs and SESCCs present a brownish area under NBI. The sensitivity of NBI for the detection of SESCCs and SPSCCs has been reported to be over 80% [4,8,11]. Thus, NBI has been an essential tool for routinely performed endoscopies for decades [12]. 

Recently, texture and color enhancement imaging (TXI) has been developed as a new image-enhanced endoscopy technology [13,14]. TXI maintains more natural color imaging than NBI and shows the appearance of WLI. There are two modes of TXI in terms of the enhancement factors: TXI mode2 enhances texture and brightness, and TXI mode1 also enhances color. Under TXI mode1, the color contrast between red and white is greater than in mode2, and reddish mucosa appears redder. Thus, the color and morphological changes in SPSCCs or SESCCs are thought to be enhanced under TXI mode1, and the visibility of SCCs is believed to have improved. However, to the best of our knowledge, no study has reported the usability of TXI for the detection of SPSCCs and SESCCs. This study aims to show the benefits of TXI for detecting SESCCs and SPSCCs compared to WLI.

## 2. Materials and Methods

### 2.1. Patients and Study Design

This retrospective trial used endoscopic images collected in a prospective study (UMIN000042428). In summary, patients with superficial neoplasms, including SCC or an intraepithelial neoplasia (IN) of the pharynx or esophagus, were prospectively recruited. The patients underwent endoscopy, and we analyzed the endoscopic images. The prospective trial was conducted in a single center between 1 November 2020 and 31 July 2021. The exclusion criteria in the prospective trial were as follows: (1) patients aged < 20 years; (2) patients who received radiotherapy where the superficial neoplasm was located; (3) patients who were pregnant; and (4) patients who were not allowed to undergo a biopsy. The Institutional Review Board at the Jikei University School of Medicine approved this study (IRB No. 31-109) on 14 September 2020.

### 2.2. Endoscopy System and Setting

A newly developed video-endoscopy system (EVIS X1, Olympus, Tokyo, Japan) and high-definition endoscopes (GIF-EZ1200 or GIF-XZ1500, Olympus) were used. This system can promptly change image modalities (WLI, TXI mode1, TXI mode2, and NBI) by the push of a button on the scope holder. The structural enhancement function was set to A5 in the WLI; TXI mode1; and mode2, and to A8 in the NBI.

### 2.3. Texture and Color Enhancement Imaging (TXI)

**Figure 1:**
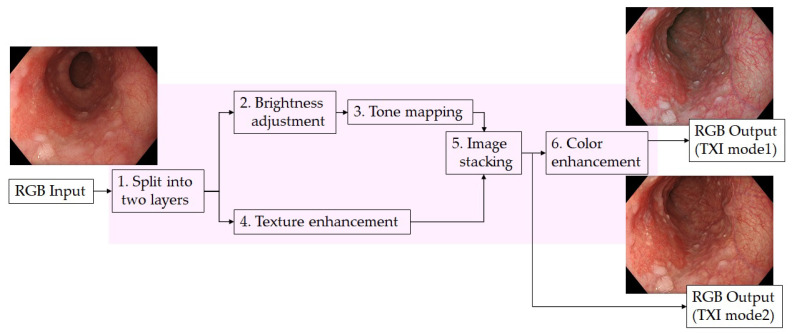
The algorithm of texture and color enhancement imaging (TXI).

TXI enhances three image factors in WLI (texture, brightness, and color) using the described algorithm (Figure 1) [13]. In short, the WLI is split into the base and detail layers. Then, the brightness in the dark regions is adjusted using the base layer, and the texture is enhanced by using the detail layer. The TXI mode2 image is obtained by stacking these layers. Color tone enhancement, which expands the color difference between white and red, is added only to TXI mode1.

### 2.4. Endoscopic Procedure and Collecting Endoscopic Images

**Figure 2:**
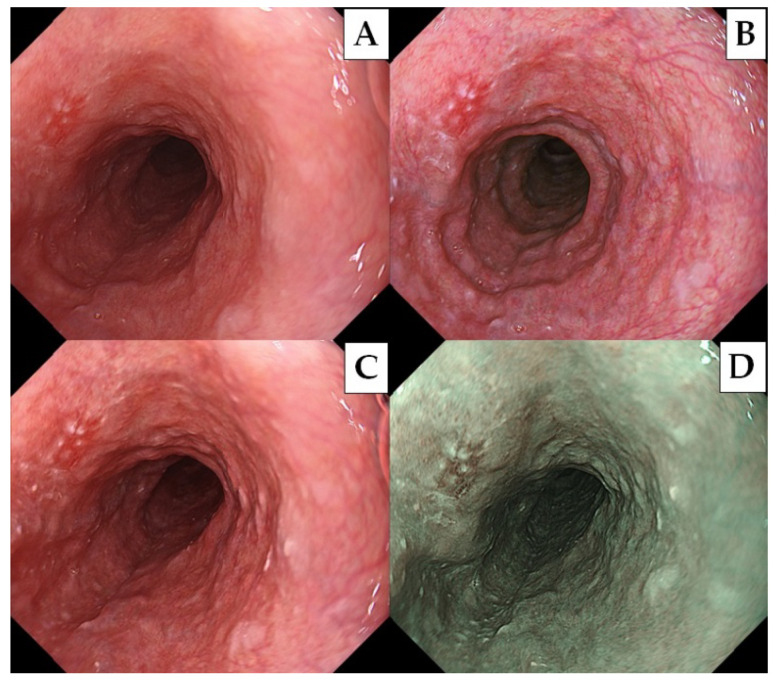
A demonstrative case of superficial esophageal cancer. The lesion was observed by (**A**) WLI, (**B**) TXI mode1, (**C**) TXI mode 2, and (**D**) NBI. The histology obtained from the endoscopic submucosal dissection revealed squamous cell carcinoma invading the lamina propria mucosa.

All endoscopic examinations were performed under conscious sedation using intravenous midazolam (2–5 mg, Maruishi Pharmaceutical Co., Ltd. Osaka, Japan) and pethidine hydrochloride (35 mg, Pethidine, Takeda Pharmaceutical Co., Osaka, Japan). Patients were initially examined using NBI. When an examiner found an SCC suspicious lesion, the examiner changed the modalities and stored the endoscopic images under the same endoscopic view (Figure 2). We defined SCC suspicious lesions as well-demarcated brownish areas > 5 mm in diameter, or depressed/elevated lesions under NBI in the non-magnification mode [8]. All procedures were performed by a single expert endoscopist (A.D.), who was certified by the board of Japan Gastroenterological Endoscopy Society and had experience with more than 1000 cases of endoscopic evaluation for staging esophageal cancer. Histology was performed by biopsy or the endoscopic submucosal dissection of all SCC suspicious lesions.

### 2.5. Color Analysis

**Figure 3:**
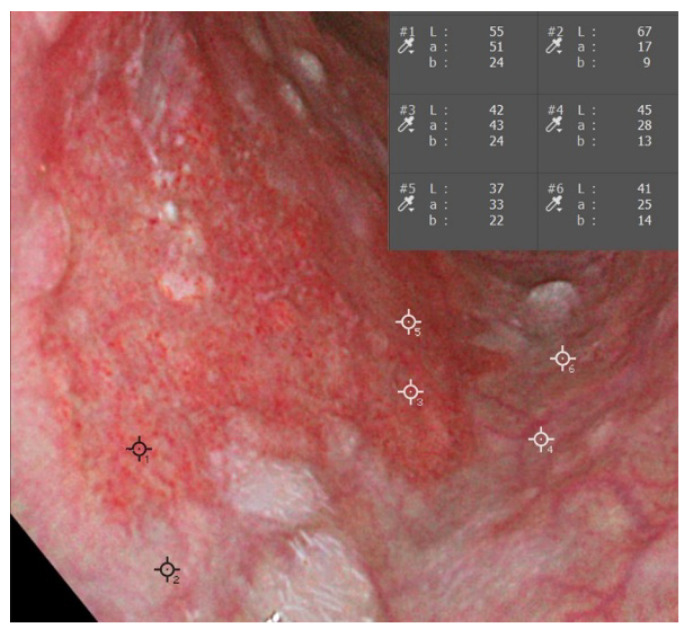
Color analysis was conducted using computer software.

We evaluated the color values based on the Commission Internationale d’Eclairage (CIE) L*a*b* color space developed in 1976 by the International Commission on Illumination [15]. The color value was shown with three-dimensional color parameters: L* (black to white, range 0 to +100); a* (green to red, range −128 to +127); and b* (blue to yellow, range −128 to +127). Once a region of interest (ROI) consisted of 121 pixels (11 × 11 pixels), it was set on the endoscopic image using Adobe Photoshop 2021 version: 22.4.2 (Adobe Systems Inc., San Jose, California, USA). The median L*a*b* value was automatically calculated (Figure 3). We defined the color values of the lesion as L*l; a*l; and b*l, and the surrounding background mucosa as L*s, a*s, and b*s. The value difference was calculated as follows: ΔL = L*l − L*s; Δa = a*l − a*s; and Δb = b*l − b*s. The color difference between the lesion and the surrounding background mucosa (ΔE) was calculated using the following formula: ΔE = √(ΔL^2^ + Δa^2^ + Δb^2^) [16]. We selected three pairs of ROIs from the lesion and the surrounding mucosa, and the mean ΔE was calculated.

A region of interest (ROI) was selected in the lesion (#1) and surrounding mucosa (#2) on the endoscopic image. The color value based on the L*a*b* color space was automatically calculated. We selected three pairs of ROIs for each lesion.

### 2.6. Outcomes

The primary endpoint was the mean ΔE for each modality. Each value was compared to that of the WLI. The secondary endpoints were comparing the ΔE by lesion location, histology, macroscopic type, and lesion size. Six endoscopists evaluated the visibility of each modality.

### 2.7. Pathologic Diagnosis

A histological diagnosis was established using biopsied or endoscopically resected specimens. Each specimen was graded as follows: no neoplasia, indefinite for neoplasia (IDN), intraepithelial neoplasia (IN), and SCC [17]. IN was also divided into two grades according to the Japanese Classification of Esophageal Cancer, 10th edition: low-grade intraepithelial neoplasia (LGIN) and high-grade intraepithelial neoplasia (HGIN) [18]. 

### 2.8. The Evaluation of Endoscopic Images

Endoscopic images, including WLI, TXI mode1, TXI mode2, and NBI (Figure 2), were used to evaluate visibility. The images were shown on a 32-inch monitor with 4K resolution clinically used for endoscopy. The histological diagnoses were blinded. Three expert endoscopists who performed > 10,000 esophagogastroduodenoscopies and three non-expert endoscopists who performed < 300 esophagogastroduodenoscopies evaluated the visibility. The endoscopists scored the visibility of the lesions on each image obtained via TXI mode1; TXI mode2; and NBI, compared to that of the WLI, according to the following scale: +1 (improved visibility of lesion), 0 (unchanged visibility of lesion), and −1 (worsened visibility of lesion) [10]. 

### 2.9. Statistical Analysis

All statistical analyses were performed using SAS (version 9.4, SAS Institute, Cary, NC, USA). The quantitative data collected of the color differences were compared using the Wilcoxon signed-rank sum test. The visibility scores were compared using a t-test or one-way ANOVA with Tukey’s post hoc test. The proportions of people who reported an improvement or a decline were compared using Fisher’s exact test. The statistical significance was set at *p* < 0.05.

## 3. Results

A total of 59 suspicious SCC lesions from 30 patients were analyzed. The lesion characteristics are listed in Table 1. The histology revealed SCCs in 24 lesions, INs in 27 lesions, and other diagnoses (no neoplasia/IDNs) in 8 lesions. Macroscopically, 8 lesions (13.6%) were type 0-IIa, 35 lesions (59.3%) were type 0-IIb, and 17 lesions (28.8%) were type 0-IIc. 

**Figure 4:**
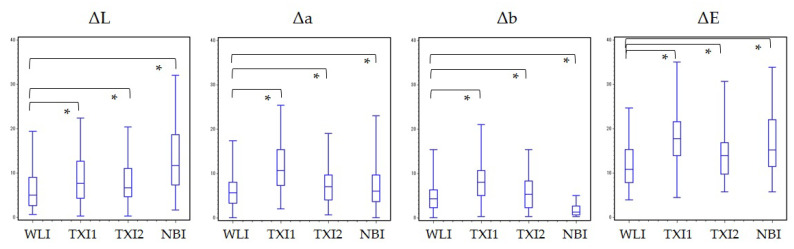
The result of the color difference analysis. * means *p* < 0.001.

In the color difference analysis, the values of ΔL; Δa; and Δb in TXI mode1, TXI mode2, and NBI were higher than those in WLI, except for Δb in NBI. There were significant differences between the groups (*p* < 0.001, Table 2 and Figure 4). The mean ΔE values in WLI, TXI mode1, TXI mode2, and NBI were 11.6; 18.6; 14.3; and 17.2, respectively, and the ΔE values of TXI mode1, TXI mode2, and NBI were significantly higher than those of the WLI (*p* < 0.001). the subgroup analysis based on location; histology; type; and size also revealed significant differences in the ΔE values of TXI mode1, TXI mode2, and NBI, compared to WLI (Table 3). 

The values of ΔL, Δa, and Δb in TXI mode1, TXI mode2, and NBI were higher than those in the WLI, except for Δb in NBI. There were significant differences between the groups (* *p* < 0.001). The mean ΔE values in the WLI, TXI mode1, TXI mode2, and NBI were 11.6; 18.6; 14.3; and 17.2, respectively, and the ΔE values of TXI mode1, TXI mode2, and NBI were significantly higher than those of the WLI (* *p* < 0.001).

The results of the visibility assessment are presented in Table 4. In TXI mode1, no lesion was evaluated as having worse visibility, and 62.5% (37/59) had improved visibility, as assessed by more than half of the endoscopists. On the other hand, in NBI, two lesions had worse visibility, and 88.1% (52/59) of the lesions had improved visibility, as assessed by more than half of the endoscopists. There were significant differences in the mean visibility scores between the three modalities, for both experts and non-experts (*p* < 0.001). With the post hoc test, there were significant differences between all pairs of modalities (mean visibility score in TXI mode1 vs. in NBI = 0.68 vs. 0.92; in TXI mode1 vs. in TXI mode2 = 0.68 vs. 0.49; and in TXI mode2 vs. in NBI = 0.49 vs. 0.92) for the experts. There were also significant differences between all pairs of modalities (mean visibility score in TXI mode1 vs. in NBI = 0.58 vs. 0.75; in TXI mode1 vs. in TXI mode2 = 0.58 vs. 0.31; and in TXI mode2 vs. in NBI = 0.31 vs. 0.75) for the non-experts. The mean visibility scores in TXI mode2 and the NBI were significantly higher for experts than for non-experts.

The results of the subgroup analyses are summarized in Table 5. The histology affected the visibility scores in TXI mode1 and the NBI, and higher visibility scores tended to be malignancies. However, there was no correlation between the visibility score and other factors, including location (pharynx vs. esophagus), macroscopic type (IIb vs. IIa or IIc), and size (<10 mm vs. ≥10 mm). 

## 4. Discussion

This study demonstrated that TXI, which enhanced the brightness, structure, and color of the images, expanded the ΔE between the inside lesion and surrounding mucosa and improved the visibility of the SCC suspicious lesions, compared to the WLI in the pharynx or esophagus. In particular, TXI mode1 emphasizes the color tone, so the ΔE value is highest in other modalities, and the visibility of the lesions is improved. In fact, in TXI mode1, there were no lesions whose visibility was worse than the WLI. TXI has been developed as a new digital IEE [6] and has become a new reliable modality for upper gastrointestinal screening endoscopy.

In a previous study, the ΔE value between esophageal cancer and the surrounding mucosa was examined. There was no correlation between ΔE and the invasion depth of SESCC in the WLI [7]. When INs and non-neoplastic lesions other than SCC were examined in this study, the ΔE value increased even in the WLI. The ΔE values increased according to LGIN, HGIN, and SCC. Therefore, it was demonstrated that it is appropriate to pay attention to the color difference to detect more malignant lesions. Compared to WLI, TXI amplifies the ΔE value by approximately 1.5 times, and the more significant ΔE value improved the visibility of the lesions. We examined the features of lesions in which the ΔE value was amplified by the TXI and found that the results were independent of the lesions’ characteristics, such as malignancy, macroscopic type, and lesion diameter. Thus, TXI can improve the ΔE value for any SCC suspicious lesions in the pharynx and esophagus.

The recognition of color and morphological changes is essential for early esophageal cancer detection [5,10]. Since type 0-IIb lesions are entirely flat, theoretically, only brightness should be enhanced when observed in TXI mode2 (Figure 1). On the other hand, the structure is also enhanced when type 0-IIa and -IIc lesions are observed in TXI mode2. In this study, no difference in visibility was observed between type 0-IIb, 0-IIa, and 0-IIc lesions in TXI mode2 (Table 5). The esophagus has a smooth epithelium, and morphological changes can be sufficiently detectable even with WLI using a high-definition endoscope. This suggests that enhancing the color tone improves the visibility of pharyngeal and esophageal cancers, rather than emphasizing the structure. TXI mode2 did not contain an algorithm to emphasize the color tone, and the ΔE value was improved by the value of ΔL (Table 2, Figure 4) [13]. The lower ΔE value in TXI mode2 was sufficient, but the visibility score was lower than that of TXI mode1. Therefore, TXI mode1 was considered to have a more significant benefit than TXI mode2.

Among the modalities, TXI mode1 had the highest ΔE value. On the other hand, in the evaluation of visibility, the mean visibility score of the NBI was the highest. Under NBI, an endoscope captures the reflected light emitted via the blue and green filters, and the endoscopic image turns green overall [5]. This greenish image causes the lowest Δb, following a lower ΔE than that in TXI mode1. Dilated irregular microvessels and a high density of microvessels are seen on the surface of SPSCCs and SESCCs [19,20], and these microvessels result in redder lesions under the WLI. When we observed such lesions under non-magnifying NBI, the lesion turned brownish and consisted of irregular microvessels. Thus, we can recognize SCC not only by color changes but also by microvessel patterns when using high-definition endoscopy. The software calculated the L*a*b* value as the average value of 121 pixels in the ROI. The image in the ROI was changed to a uniform color, and the ΔE value was calculated [16]. Under endoscopy, endoscopists should recognize a lesion not only by the color change but also by the microvessel pattern in the pharynx and esophagus. Therefore, the NBI showed a moderate increase in the ΔE value and the highest visibility score. The higher detection rate of NBI for SPSCCs and SESCCs has already been reported, and NBI has become a reliable tool for upper gastrointestinal screening endoscopy [4,8,11,12]. It is unclear whether TXI mode1 is sufficiently effective in detecting SPSCCs and SESCCs in clinical practice because, in this study, it is an evaluation method that has been compared with WLI. In the future, it will be necessary to evaluate the detectability (sensitivity) of lesions in each modality and to conduct prospective controlled trials.

The benefits of TXI are that endoscopists can perform an endoscopy with a WLI-like tone and detect suspicious SCC lesions by solely paying attention to a color change. The WLI-like tone should be preferable, especially for endoscopists who have less experience with endoscopy or with detecting SPSCCs or SESCCs using NBI. In this study, experts tended to have higher NBI visibility ratings than non-experts. The higher score in NBI may reflect the need to familiarize them with the NBI endoscopic images to detect lesions under NBI [21]. On the other hand, the visibility evaluation results of TXI mode1 showed no differences between experts and non-experts; even the trainees could visually detect the lesions in the same manner as the experts. 

This study had some limitations. First, this study included a small number of lesions, which should have caused a negative result in the subgroup analysis. An analysis with a more significant number of lesions can reveal lesions that are difficult to detect using TXI mode1. Second, the field of the endoscopic view was not always consistent due to peristalsis, especially for lesions in the esophagus. This problem may have influenced the evaluation of the ΔE value and visibility. A prospective trial with real-time visibility evaluations is required to elucidate this issue.

## 5. Conclusions

TXI mode1 enhanced color changes and improved the visibility of SCC suspicious lesions in the pharynx and esophagus compared to the WLI. This newly developed digital IEE can compensate for the weaknesses of WLI and NBI and can also become a reliable modality for detecting superficial SCCs during upper gastrointestinal endoscopy.

## Figures and Tables

**Table 1:** Demographics of the squamous cell carcinoma suspicious lesions.

Characteristic		*N*	Rate
Location	Pharynx	15	25.4%
	Esophagus	44	74.6%
Histology	SCC	24	40.7%
	HGIN	11	18.6%
	LGIN	16	27.1%
	Indefinite for neoplasia, no neoplasia	8	13.6%
Type	IIb	24	40.7%
	IIa or IIc	35	59.3%
Size	<10 mm	27	45.8%
	≥10 mm	32	54.2%

**Table 2:** The mean ΔE of WLI, TXI mode1, TXI mode2, and NBI.

	WLI	TXI Mode1	TXI Mode2	NBI
ΔL	6.2(4.4)	8.7 (5.4)	8.1 (5.1)	13.2 (7.6)
*p* (vs. WLI) *		<0.001	<0.001	<0.001
Δa	5.7 (3.7)	10.8 (5.4)	7.2 (3.9)	6.9 (4.5)
*p* (vs. WLI )*		<0.001	<0.001	<0.001
Δb	4.8 (3.4)	8.3 (4.8)	5.6 (3.4)	1.8 (1.3)
*p* (vs. WLI) *		<0.001	<0.001	<0.001
ΔE	11.6 (4.8)	18.6 (6.8)	14.3 (5.5)	17.2 (7.3)
*p* (vs. WLI) *		<0.001	<0.001	<0.001

Values are presented as mean (SD). * Wilcoxon signed-rank sum test.

**Table 3:** The results of the comparisons of the color difference by location, histology, macroscopic type, and size.

		WLI	TXI Mode1	TXI Mode2	NBI
Location	Pharynx	10.4(3.8)	16.2(5.6)	13.3(4.8)	13.8(5.9)
	*p* (vs. WLI) *		<0.001	<0.001	<0.001
	Esophagus	11.9(5.1)	19.4(7.0)	14.7(5.8)	18.4(7.4)
	*p* (vs. WLI) *		<0.001	<0.001	<0.001
Histology	SCC	12.6(5.4)	19.8(5.6)	15.2(4.9)	19.2(7.1)
	*p* (vs. WLI) *		<0.001	<0.001	<0.001
	HGIN	11.4(3.1)	19.6(7.2)	14.7(2.7)	15.4(4.5)
	*p* (vs. WLI) *		0.001	0.001	0.001
	LGIN	10.0(3.4)	16.0(5.7)	12.1(4.4)	14.7(5.4)
	*p* (vs. WLI) *		0.001	0.001	0.001
	Indefinite for neoplasia, no neoplasia	11.6(6.8)	18.6(10.8)	15.7(10.3)	18.8(12.0)
	*p* (vs. WLI) *		0.008	0.008	0.008
Type	IIb	10.6(4.4)	18.4(6.8)	14.2(5.0)	17.1(7.3)
	*p* (vs. WLI) *		<0.001	<0.001	<0.001
	IIa or IIc	12.9(5.1)	18.9(6.9)	14.6(6.4)	17.4(7.4)
	*p* (vs. WLI) *		<0.001	<0.001	<0.001
Size	<10 mm	11.0(4.3)	18.6(8.0)	14.5(6.3)	17.6(7.6)
	*p* (vs. WLI) *		<0.001	<0.001	<0.001
	≥10 mm	12.2(5.3)	18.5(5.1)	14.2(4.6)	16.7(7.0)
	*p* (vs. WLI) *		<0.001	<0.001	<0.001

Values are presented as mean (SD). * Wilcoxon signed-rank sum test.

**Table 4:** The results of the visibility assessments.

		Experts		Non-Experts		*p*
TXI mode1 visibility	Score, mean (SD) *	0.68 (0.34)		0.58 (0.33)		0.072
	Number reporting an improvement					
	0	6	10.2%	6	10.2%	0.229
	1	11	18.6%	17	28.8%	
	2	18	30.5%	22	37.3%	
	3	24	40.7%	14	23.7%	
	Number reporting a decline					
	0	59	100.0%	59	100.0%	-
	1	0	0.0%	0	0.0%	
	2	0	0.0%	0	0.0%	
	3	0	0.0%	0	0.0%	
TXI mode2 visibility	Score, mean (SD) *	0.49 (0.37)		0.31 (0.35)		<0.001
	Number reporting an improvement					
	0	10	16.9%	23	39.0%	0.022
	1	20	33.9%	21	35.6%	
	2	16	27.1%	8	13.6%	
	3	13	22.0%	7	11.9%	
	Number reporting a decline					
	0	56	94.9%	57	96.6%	1.000
	1	3	5.1%	2	3.4%	
	2	0	0.0%	0	0.0%	
	3	0	0.0%	0	0.0%	
NBI visibility	Score, mean (SD) *	0.92 (0.17)		0.75 (0.31)		<0.001
	Number reporting an improvement					
	0	0	0.0%	2	3.4%	0.005
	1	2	3.4%	10	16.9%	
	2	12	20.3%	18	30.5%	
	3	45	76.3%	29	49.2%	
	Number reporting a decline					
	0	59	100.0%	57	96.6%	0.496
	1	0	0.0%	2	3.4%	
	2	0	0.0%	0	0.0%	
	3	0	0.0%	0	0.0%	

* Comparison using paired one-way ANOVA.

**Table 5:** The results of the subgroup analysis for visibility scores by location, histology, macroscopic type, and size.

		Experts			Non-Experts		
		TXI mode1 visibility	TXI mode2 visibility	NBI visibility	TXI mode1 visibility	TXI mode2 visibility	NBI visibility
Location	Pharynx	0.68(0.29)	0.48(0.34)	0.92(0.14)	0.51(0.32)	0.23(0.29)	0.63(0.32)
	Esophagus	0.68(0.36)	0.50(0.38)	0.91(0.18)	0.61(0.33)	0.34(0.37)	0.79(0.31)
	*p*	0.962	0.857	0.901	0.329	0.330	0.089
Histology	SCC	0.77(0.34)	0.54(0.38)	0.93(0.13)	0.68(0.29)	0.34(0.38)	0.83(0.24)
	HGIN	0.67(0.34)	0.63(0.33)	0.97(0.09)	0.57(0.35)	0.39(0.42)	0.67(0.27)
	LGIN	0.72(0.30)	0.44(0.39)	0.94(0.19)	0.50(0.31)	0.24(0.28)	0.82(0.30)
	No neoplasia, indefinite for neoplasia	0.33(0.27)	0.29(0.29)	0.76(0.24)	0.50(0.41)	0.25(0.31)	0.46(0.44)
	*p*	0.013	0.202	0.036	0.328	0.648	0.019
Type	IIb	0.77(0.26)	0.53(0.34)	0.92(0.17)	0.64(0.32)	0.35(0.33)	0.73(0.31)
	IIa or IIc	0.61(0.38)	0.47(0.39)	0.91(0.17)	0.55(0.33)	0.28(0.37)	0.76(0.32)
	p	0.060	0.644	0.835	0.306	0.475	0.678
Size	<10 mm	0.73(0.33)	0.50(0.37)	0.91(0.14)	0.66(0.31)	0.30(0.25)	0.82(0.23)
	≥10 mm	0.63(0.35)	0.49(0.37)	0.92(0.19)	0.52(0.33)	0.32(0.36)	0.68(0.36)
	*p*	0.244	0.923	0.864	0.090	0.898	0.076

Values are presented as the mean (SD) of the visibility scores. They were compared using a *t*-test or one-way analysis of variance.

## Data Availability

The data that support the findings of this study are available from the corresponding author upon reasonable request.
